# New York Palaces

**Published:** 1871-08

**Authors:** 


### HALL'S

### JOURNAL OF HEALTH.

Vol. XVIII. — AUGUST, 1871. — No. VIII.

### NEW YORK PALACES.

Many a private dwelling in New York city is more imposing in appearance than the "palace" of St. James, where the predecessors of Queen Victoria lived so long in royal splendor, and yet these same palatial residences of the merchant princes of the great metropolis of the Western world sadly lack one of the very first and greatest of all domestic luxuries, —

### WATER.

"Water, water, everywhere, and not a drop to drink," cries the dying castaway at sea. Notwithstanding the millions of money expended on the delicious "Croton," it scarcely rises to the second floor of the grandest mansions on aristocratic "Murray Hill;" hence many a two-story brick house in the lower parts of the town boasts of the privilege of a plenty of Croton, day and night, summer and winter, from cellar to attic, and in this has the inside track of the lordly "brown stone front." The advantage of an abundant supply of water in every chamber, for comfort, enjoyment, and health, can scarcely be overestimated, and yet the cost and trouble of securing it has been so great that many persons have abandoned all hope of it, and have adopted the old-time plan of having a basin or pitcher of water taken up every night, and as often during the day as may be required; and the result is, arising from the forgetfulness or inattention of servants, an amount of calling, and waiting, and bell-ringing, and annoyance, and that daily, which is almost incalculable. A guest gets up early, so as not to keep the family waiting for breakfast. He finds there is no water in his room. He does not like to call for it, and waits, in the vain hope that a servant will come along soon; time passes, breakfast is announced, and there he is, half dressed, sitting on the edge of his bed, in his shirt sleeves, in utter despair, helplessness, and — shall it be said? — rage; the reader is left to complete the picture, made up of impatience and apologies and mortifications, ending in the "rating" of servants; they in turn venting maledictions on the man who was the innocent cause of all, winding up with one earnest prayer at last, "Wish you would stay at home next time."

Various plans have been devised for constructing "tanks" on the tops of houses or the upper floors; but tanks are useless unless they are kept full of water, which must be conveyed to them by artificial means. Steam pumps are costly and troublesome — few can afford an "engineer" in their households; water rams cost several hundred dollars a year, because the greater part of the water is wasted, and the Croton department has heretofore taxed the occupant for this waste; but of late it has been determined to allow no more rams, as the waste cannot be afforded. Several hand-pumps have been devised and patented, with a tax of fifty or a hundred dollars annually; but servants will not work them, and help must be had outside every day, making quite a bill at the end of the year. But there seems to be no limit to human ingenuity, and deep-thinking. Irrepressible, practical New England, the "Hub," comes to "Gotham's" rescue. J. Claxton Wightman, Esq., of Boston, consulting city engineer, has invented and patented an

" Automatic House Tank Pump," which by the extra heat of a common range or stove " water-back," or boiler, will fill the tank at the top of any dwelling from any pipe, well, spring, or stream of water, in or near a building, without any other labor than that of turning a screw, to be let alone until the tank is filled, and then to be turned back again.

D. D. Williamson, Esq., of New York, says of it,— " It is giving me perfect satisfaction. It elevates a sufficient supply of water to the tank in the fourth story, without any special fire or care on the part of the servants. The relief from the nervous noise of the 'hydraulic pump' is a great blessing."

Another of our prominent and enterprising citizens writes: " I reside at 323 West Fifty-seventh Street, in a house which could not be bought for less than $75,000. Not a drop of water will rise above the parlor floor without forcing. There is not a house in the block which would not have one."

The *Evening Mail* says, June 17th:—

" It is hardly necessary to go into an explanation of the different causes which render the supply of Croton to the upper stories of nearly all New York houses an uncertainty; but the fact exists that with millions of dollars invested in magnificent engineering works for supplying water to the city, seven out of ten houses can receive water by the Croton pressure no higher than the second floor, and all the sleeping rooms, including the baths, water-closets etc., have to be supplied from water pumped up to a tank in the attic story.

" This pumping, whether performed by hand or an engine, is not only an insufferable nuisance, but attended by an expense that forms quite a serious item in the domestic economy. For the last nine or ten months, an *Automatic House Tank Pump*, invented and patented by Messrs. J. C. and H. M. Wightman, the latter gentleman Assistant Chief Engineer of the city of Boston, and constructing engineer of the Chestnut Hill Reservoir, near that city, has been tested in several of our Murray Hill residences, performing, without extra care, expense, or waste of water, the duty of supplying the upper stories with water. It has not only been entirely successful, but has elicited the highest encomiums from those who have used it. It has been introduced to the notice of the public through the well-known firm of Wm. S. Carr & Co., Nos. 106, 108, and 110 Centre Street, and also tested by them in their warerooms, where it is on exhibition, and, after examination by the proper officers of the Croton Department, has been passed upon as being of such a nature as to demand no extra charge for its use.

It is not too much to say that no invention has ever been brought to this city that is of so much value in the domestic affairs of our citizens as this, as it is the only self-operating tank pump that requires no management, and for the use of which no extra charge is made by the authorities."

No private residence is complete which has not an abundant supply of water for every floor, for it is essential to comfort, cleanliness, and health, as every intelligent reader will admit.

